# ZebIAT, an image analysis tool for registering zebrafish embryos and quantifying cancer metastasis

**DOI:** 10.1186/1471-2105-14-S10-S5

**Published:** 2013-08-12

**Authors:** Teppo Annila, Eero Lihavainen, Ines J Marques, Darren R Williams, Olli Yli-Harja, Andre Ribeiro

**Affiliations:** 1 Department of Signal Processing, Tampere University of Technology, Tampere, 33720, Finland; 2 Cardiovascular Development and Repair, Centro Nacional de Investigacoes Cardiovasculares, Madrid, 28029, Spain; 3 New Drug Targets Laboratory, School of Life Sciences, Gwangju Institute of Science and Technology, Gwangju, 500-712, Republic of Korea

## Abstract

**Background:**

Zebrafish embryos have recently been established as a xenotransplantation model of the metastatic behaviour of primary human tumours. Current tools for automated data extraction from the microscope images are restrictive concerning the developmental stage of the embryos, usually require laborious manual image preprocessing, and, in general, cannot characterize the metastasis as a function of the internal organs.

**Methods:**

We present a tool, ZebIAT, that allows both automatic or semi-automatic registration of the outer contour and inner organs of zebrafish embryos. ZebIAT provides a registration at different stages of development and an automatic analysis of cancer metastasis per organ, thus allowing to study cancer progression. The semi-automation relies on a graphical user interface.

**Results:**

We quantified the performance of the registration method, and found it to be accurate, except in some of the smallest organs. Our results show that the accuracy of registering small organs can be improved by introducing few manual corrections. We also demonstrate the applicability of the tool to studies of cancer progression.

**Conclusions:**

ZebIAT offers major improvement relative to previous tools by allowing for an analysis on a per-organ or region basis. It should be of use in high-throughput studies of cancer metastasis in zebrafish embryos.

## Introduction

Zebrafish is becoming a widely used model organism in biomedical research due to a number of features that are useful in the study of cancer progression. These include rapid development and transparency of the embryos, which allows in vivo imaging of internal organs at different stages of development. Moreover, their maintenance costs are low when compared to other model organisms and there is little variability in the morphology of embryos. Finally, zebrafish reproduce at a fast rate and can be maintained in small volumes of water [1].

This organism has recently become a model organism in studies of cancer formation, cell migration and invasion, as well as metastasis formation [1-3], among other. A zebrafish model has also been validated for anti-cancer drug screening [4]. These studies rely heavily on microscope imaging and require the analysis of a large number of images. Given that manual image analysis is often cumbersome and subjective, there is a need for automating as many steps of the data analysis as possible. A particularly important and cumbersome step in such studies is registration. Its goal is to allow the use of the same coordinate system in the analysis of all images, which is necessary for combining and comparing measurements in many individuals.

Recent studies addressed the problem of zebrafish registration (for a review, see [5]). In [6], a novel embryo's detection, registration and segmentation tool was proposed to study gene expression at an early development stage. This method is applicable to prim-20 and long-pec stages alone as the shape model of the segmentation relies on features of the embryos' outline that exist only in these stages. Recently, an assay for analyzing human cancer dissemination within zebrafish was proposed [7]. The fish were aligned horizontally and cancer spots were segmented. Their dissemination was quantified by measuring distances of cancer cells to the injection site. Unfortunately, it cannot be used to segment internal organs. Another automatic segmentation and registration tool was proposed in [8], which focuses solely on the segmentation and registration of the caudal vasculature.

Here, we propose a novel tool, ZebIAT, that automatically aligns the organs of zebrafish embryos and other regions of interest with a landmark-based thin plate splines (TPS) registration method. Its main application is the automated analysis of cancer cells migration and invasion to the organs of the embryos. In contrast to previous methods, ZebIAT works with zebrafish embryos with development stages between 2 and 5 days post fertilization (dpf) and registers all major organs. The registration can be performed using images from either a fluorescence or a differential interference contrast (DIC) microscope. Finally, we show how the manual adjustment assists the registration of the inner organs.

ZebIAT is implemented in MATLAB and is available at http://www.cs.tut.fi/%7Eannilat/zebratool/. A User's manual is also available online.

## Material and methods

In this section, we describe the methods employed by ZebIAT. A detailed description of how to use ZebIAT is provided in the User's Manual (supplementary material).

### Imaging of zebrafish embryos

We use images partially used in [1]. The experiments conducted to obtain them are described in [1]. Here, we describe briefly the steps most relevant to the present study.

Pancreatic human tumor cells were stained with CM-Dil (red fluorescence, Vybrant, Invitrogen) and injected in larvae of Tg(fli1:GFP) zebrafish embryos whose vasculature expresses green fluorescent protein (GFP) throughout development, until adulthood. Tumor cells were injected at 2 dpf in the yolk of zebrafish embryos and fluorescence stereomicroscope images were taken at 0, 1, 2 and 3 days post-injection (dpi) with either a Leica DFC 420C camera attached to a Leica MZ16FA microscope or a Carl Zeiss confocal microscope. Altogether, we use 24 fluorescence images and 14 differential interference contrast (DIC) images. The former are from the maximum projection images of z-stacks obtained with the confocal microscope.

Additionally to these images, we also use of a set of DIC images acquired for, and partially used in [4], where the experiments conducted to obtain them, protocols and regulations are described.

### Zebrafish embryo registration

In general, a registration process transfers a set of data into the same coordinate system, via a transformation model [9,10]. A common transformation model is thin plate splines (TPS) [11], which has been successfully applied to remote sensing and medical images [12,13]. This model is landmark-based, i.e., it requires fixed landmark points from the target image and from the reference image, whose correspondence is established a priori. Given these, it interpolates to find a smooth mapping between the two coordinate systems [14].

**Figure 1:**
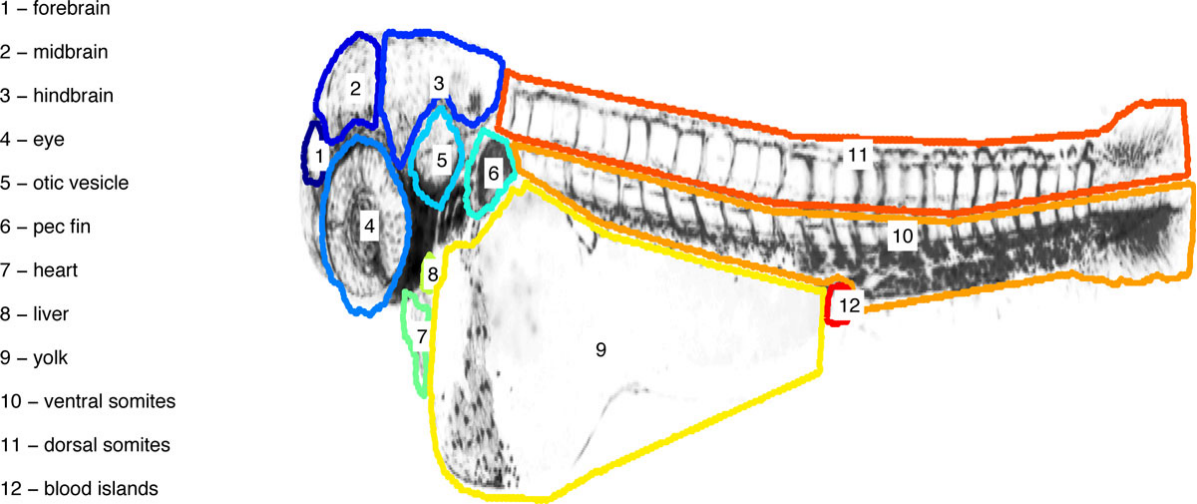
**Example of a reference embryo, with organs marked**. For easier visualization, only the borders of the masks of the organs are shown.

At this stage, one image has to be selected as the 'reference embryo'. In this image, the user manually marks the organs or other areas of interest (see User's Manual). We advise the use of different reference images for each stage of development given the rapid morphological changes. As an example, we show in (Figure 1) an example mask with several organs and areas of interest selected. ZebIAT already contains a predefined mask for each of four development stages (days 1 to 4). If the ventral and dorsal caudal regions are marked, ZebIAT has an additional option of dividing them into smaller regions. Once this procedure is complete, other fish can be automatically registered to the reference fish, and the inner organs located.

**Figure 2:**
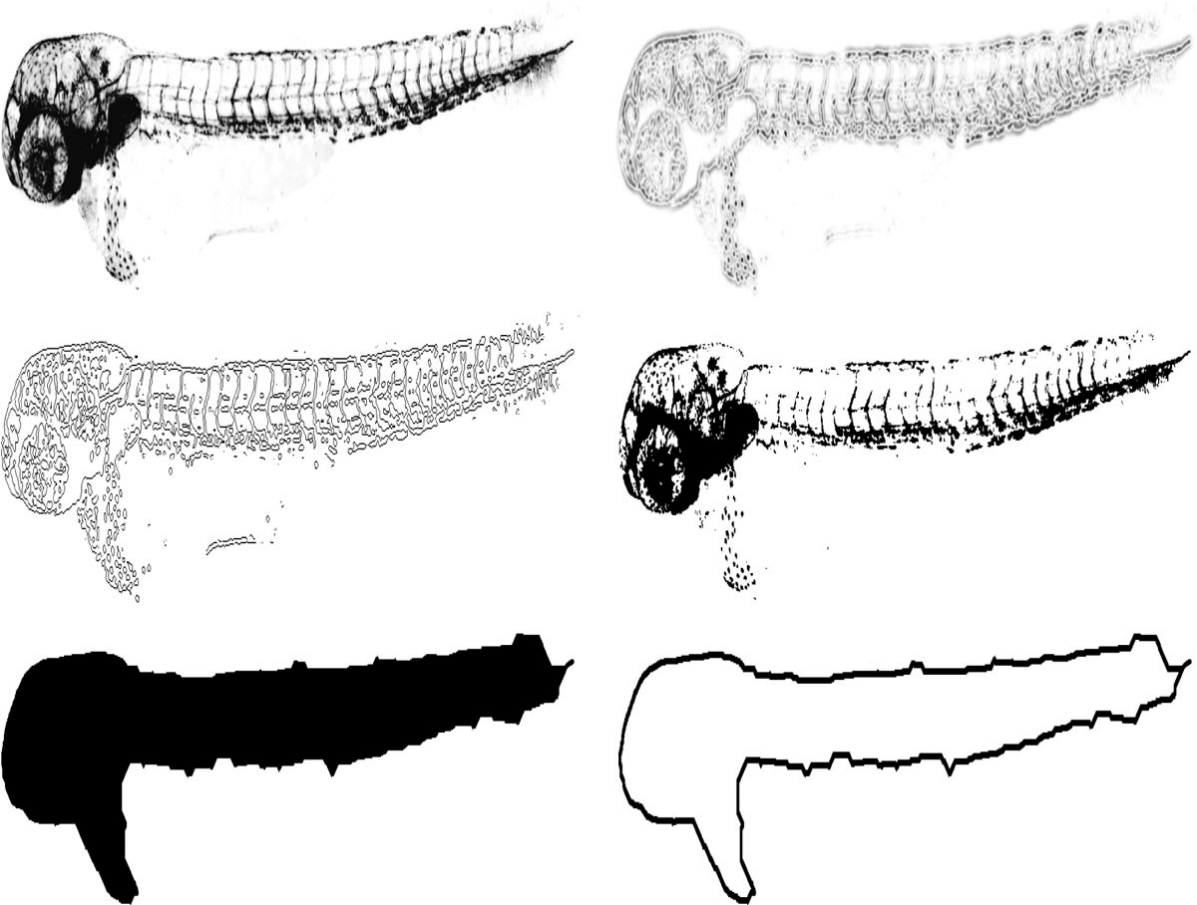
**Segmentation procedure**. Top left: Image from the green fluorescence channel. Top right: Image filtered with LoG. Middle left: Result from LoG edge detection. Middle right: Result from Otsu's threshold. Bottom left: Segmented image after all steps, including morphological closing. Bottom right: Outline of the fish. For illustration purposes, the images are presented with inverted grayscale values.

**Figure 3:**
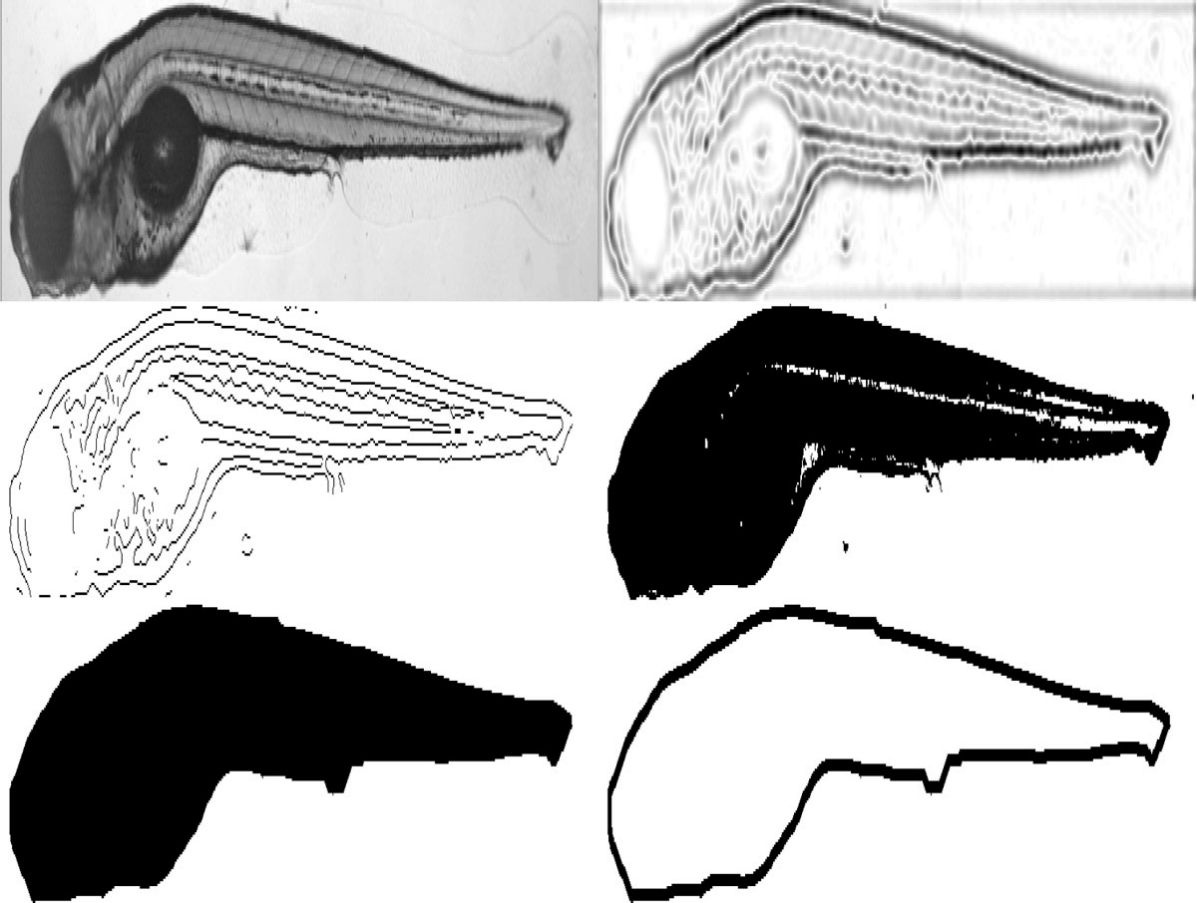
**Segmentation of a DIC image**. Top left: grayscale image with inverted grayscale values. Top right: Image filtered with LoG. Middle left: Result from LoG edge detection. Middle right: Result from Otsu's threshold. Bottom left: Segmented image after all steps, including morphological closing. Bottom right: Outline of the fish.

To perform the registration, landmarks are extracted from the images. These are obtained from the outline of the fish, since, in zebrafish, different embryos have similar shapes and outlines when at the same developmental stage. For that, the outlines of the embryo are extracted by segmentation of the image obtained from either DIC or green fluorescence channel. This segmentation is performed in several steps. First, we threshold the image using Otsu's method [15]. Since weak edges are often mapped to zero by this method, in the next step, edge detection is used. For that, we find the zero-crossings of the image filtered by a Laplacian of Gaussian (LoG) with a standard deviation of 2 and a size of 13x13, as these settings were found to suppress noise while detecting edges in fine structures such as vasculature. The result is then combined with the result from Otsu's method by a binary OR-operation. We observed that, after this step, there may still exist small gaps and holes, due to low levels of fluorescence in some areas of the vasculature. Thus, morphological closing is applied. We use a disk-shaped structuring element with a radius of 25, which was found to be large enough to fill the gaps in the vasculature. Since the mask obtained likely contains small connected components resulting from noise, we find the fish by selecting the largest connected component in the mask. Finally, the outline of the fish is obtained by a boundary tracing algorithm [16]. The segmentation steps are exemplified in Figure 2 for fluorescence images, and in Figure 3 for DIC. In the latter, the colors are inverted.

Following the segmentation, ZebIAT performs the alignment of the reference fish. This facilitates visualization and minimizes the size of stored images. To align, we seek the pairs of points in the segmented fish separated by the longest distance. These are at the head and tail. According to these, reference images are rotated so that the fish are oriented horizontally.

**Figure 4:**
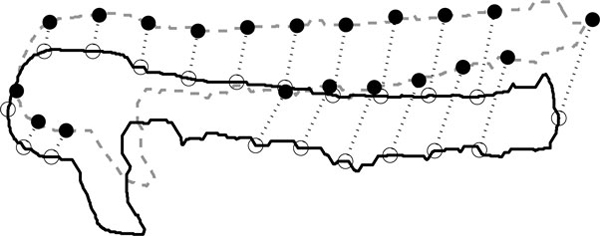
**Extracted landmarks**. The outline of the reference fish is shown as a solid line while the outline of the target fish is shown by a dashed line. Empty circles indicate the landmarks in the reference fish, while full circles indicate the landmarks in the target fish. The dotted lines indicate the correspondence between landmarks in the two fish.

Next, we use the outline to obtain landmarks automatically for both reference and non-reference fish. Once the head and tail are located, we generate new landmarks by moving along the contour and placing landmarks between them. These are introduced as follows. In general, they are equally spaced. However, in two regions, the tail and the yolk, they are not added, as these regions differ significantly in shape from one embryo to the next. The direct correspondence between landmarks of reference and non-reference fish is obtained by generating an equal amount of landmarks for both fish. The result of placing landmarks and the correspondence between reference and non-reference fish is illustrated in Figure 4. To support this process, ZebIAT has a user interface to add, remove or modify landmarks. This can be of use particularly in registering internal organs or regions of interest.

Once the landmarks are set, to find a smooth transformation, parameters of the TPS model could be fitted, for each developmental stage of the embryo, by finding the transformation *f *that minimizes the energy functional

(1) $$E_{TPS}\left(f\right)=\underset{{\mathbb{R}}^{2}}{\int }\int \left({\left(\frac{\partial^{2}f}{\partial x^{2}}\right)}^{2}+2{\left(\frac{\partial^{2}f}{\partial x\partial y}\right)}^{2}+{\left(\frac{\partial^{2}f}{\partial y^{2}}\right)}^{2}\right)dxdy,$$

while requiring that *f*(*p_i_*) = *q_i _*for all *i*, where *p_i_*, $q_{i}\in {\mathbb{R}}^{2}$ are the landmarks of the target and reference images, respectively. However, in practice it is useful to allow some degree of error in the landmark correspondences. Thus, instead of this interpolating scheme, we minimize the following functional, proposed in [13]:

(2) $$J\left(f\right)=\overset{n}{\underset{i=1}{\sum }}\frac{{\left(q_{i}-f\left(p_{i}\right)\right)}^{2}}{\sigma_{i}^{2}}+\lambda E_{TPS}\left(f\right),$$

where $\sigma_{i}^{2}$ are the weights for landmark errors and *λ *is a regularization term controlling the smoothness of the transformation. Equation 2 has a closed-form solution and can be solved in a matrix form [13]. For the regularization parameter, as in [13], we use *λ*=300. Further, we use equal scalar weights *σ_i _*= 1 to approximate the landmark errors.

**Figure 5:**
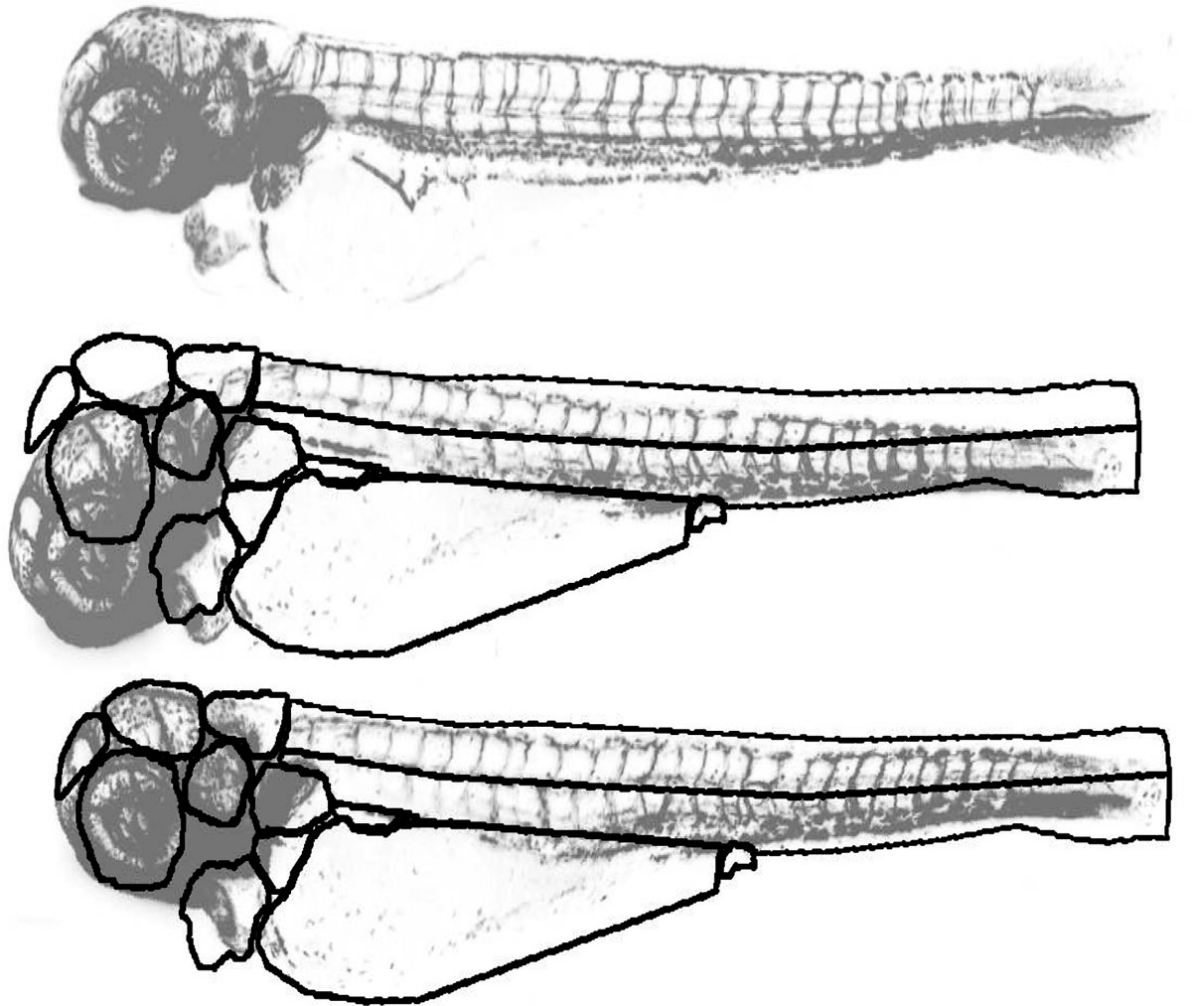
**Registration procedure**. Top: Reference fish with 1 day after injection of cancer cells. Middle: Fish to be registered. Bottom: Registered fish. For illustration purposes, the images are presented with inverted grayscale values.

Images of the reference fish, followed by the target fish, before and after the registration are shown in Figure 5. Also visible is the mask of the reference fish over the target fish, before and after registration. We used 20 landmarks, 17 selected automatically and 3 added manually.

### Spot detection

We detect fluorescently labeled cancer cells using a method of multiscale product of wavelets [17], based on the assumption that spots will be present at each level of wavelet decomposition. At the first level of decomposition, the convolution between the original image *A*_0_(*x*, *y*) and the kernel [1/16, 1/4, 3/8, 1/4, 1/16] is computed, first row-wise and then columnwise, resulting in a smoothed image *A*_1_(*x*, *y*). This procedure can be repeated recursively *J *times from smoothed approximation images. At each step *i*, the kernel is extended by padding 2*^i−1 ^*− 1 zeros between kernel coefficients. The decomposition can be formulated as

(3) $$W_{i}\left(x,y\right)=A_{i}\left(x,y\right)-A_{i-1}\left(x,y\right),0<i\leq J,$$

and the multiscale product of wavelets is then

(4) $$P_{J}\left(x,y\right)=\overset{J}{\underset{i=1}{\prod }}W_{i}\left(x,y\right).$$

We use default parameter values proposed in [17], following the implementation in [18].

**Figure 6:**
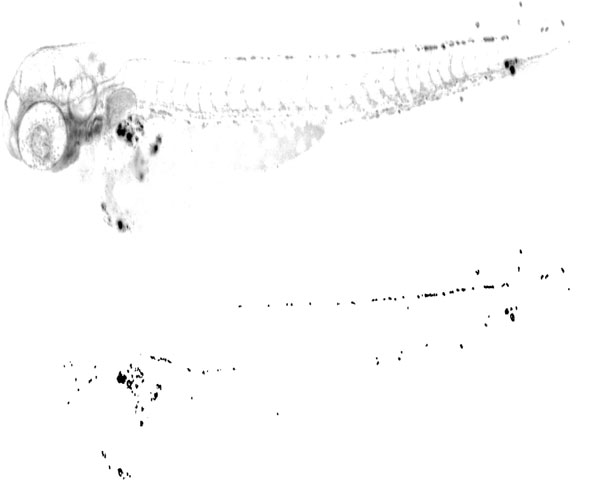
**Spot detection**. Top: red channel of the image. Bottom: segmented spots. Gamma correction was used in the two images to improve the visibility of the spots. For illustration purposes, the images are presented with inverted grayscale values.

If a spot is present at each level of decomposition, it results in a significant value of *P_J _*(*x*, *y*), which can be thresholded. If no spot is present at some level of decomposition, *P_J _*(*x*, *y*) will decrease significantly. One example of spot detection is shown in Figure 6 (bottom), which also shows the original image. As seen, the green channel leaks into red channel to some extent, but red spots are still detectable.

We found this leakage common in the fluorescence images used here. Therefore, for this specific set of images, we first removed this effect from subsequent analysis. We explain this extra procedure, as it is not included in ZebIAT but it may be of use, depending on the quality of the source images. We used an *ad-hoc *method that assumes a linear relationship between the 'green' image *G *and the 'red' image *I*:

(5) I=R+βG,

where *R *is the true red signal to be recovered, and β∈ℝ is unknown. Assuming that *R *is a random variable with zero mean and constant variance, we find an estimate $\hat{\beta }$ for *β *using Linear Least Squares. We then approximate the true red signal as $\tilde{R}\approx I-\hat{\beta }G$. Since $\tilde{R}$ is not necessarily nonnegative, we additionally set all negative values to zero.

## Results

To validate ZebIAT and assess its performance, we analyzed images of three fish at four stages of development (day 1 to 4). For each non-reference fish, we marked seven organs or areas (Figure 1). We registered these twelve images to the reference images of the corresponding developmental stages, and assessed how well the registered masks matched the masks of the reference images.

To quantify the overlap between masks, we calculated the Simpson coefficient [19], defined as the ratio between the area of the intersection of the masks and the area of the smaller mask. To capture each organ, the reference masks were drawn slightly bigger than the organ (less than 5% larger). This allows the overlap coefficient to be a measure of the percentage of the area of the registered organ that is properly masked. If the validation mask is a subset of the reference mask, the overlap coefficient equals the unity. If equal to zero, it implies that the mask does not intersect the organ in any point.

The results in Table 1 indicate that, in general, the automatic registration procedure is efficient, especially near the contour where landmarks were chosen automatically. The ventral and dorsal somites, the yolk and the brain regions are more accurately registered. However, in a few areas, such as the eye, otic vesicle and pec fin, the accuracy is lower as the automatically selected landmarks are not sufficient for ZebIAT to account for the differences between reference and non-reference fish.

**Table 1:** Overlap coefficient, automatic method

Region	Day 0	Day 1	Day 2	Day 3	Mean
Brain regions	0.88	0.88	0.90	0.84	0.87
Eye	0.76	0.87	0.75	0.89	0.82
Otic vesicle	0.71	0.65	0.64	0.84	0.71
Pec fin	0.60	0.62	0.45	0.86	0.63
Yolk	0.96	0.94	0.81	0.92	0.91
Ventral somites	0.91	0.84	0.90	0.94	0.90
Dorsal somites	0.95	0.94	0.94	0.88	0.93

To improve the registration accuracy in these areas, we next manually added three landmarks: one in the eye, one in the otic vesicle and one in the pec fin. These were added approximately in the center of each of these organs. Results with the 17 automatically chosen landmarks and the 3 manually added landmarks are shown in Table 2.

**Table 2:** Overlap coefficient.

Region	Day 0	Day 1	Day 2	Day 3	Mean
Brain regions	0.91	0.88	0.83	0.86	0.87
Eye	0.94	0.97	0.93	0.96	0.95
Otic vesicle	0.96	0.90	0.90	0.91	0.92
Pec fin	0.92	0.96	0.89	0.96	0.93
Yolk	0.94	0.97	0.95	0.96	0.95
Ventral somites	0.90	0.90	0.86	0.95	0.90
Dorsal somites	0.98	0.99	0.92	0.92	0.95

17 landmarks were chosen automatically and 3 were manually chosen.

From Table 2 one observes a mean accuracy of 90% or higher given the manually added landmarks. These, in general, not only improved significantly the accuracy in those areas where it was lower, but also slightly improved the accuracy in other areas (except in rare cases, where a small decrease was observed). Additional manual landmarks would further improve the results, with decreasing significance. We note that, in some cases, the brain region was susceptible to error due to the angle of rotation of the fish relative to its major axis, which rendered areas of the brain invisible.

**Figure 7:**
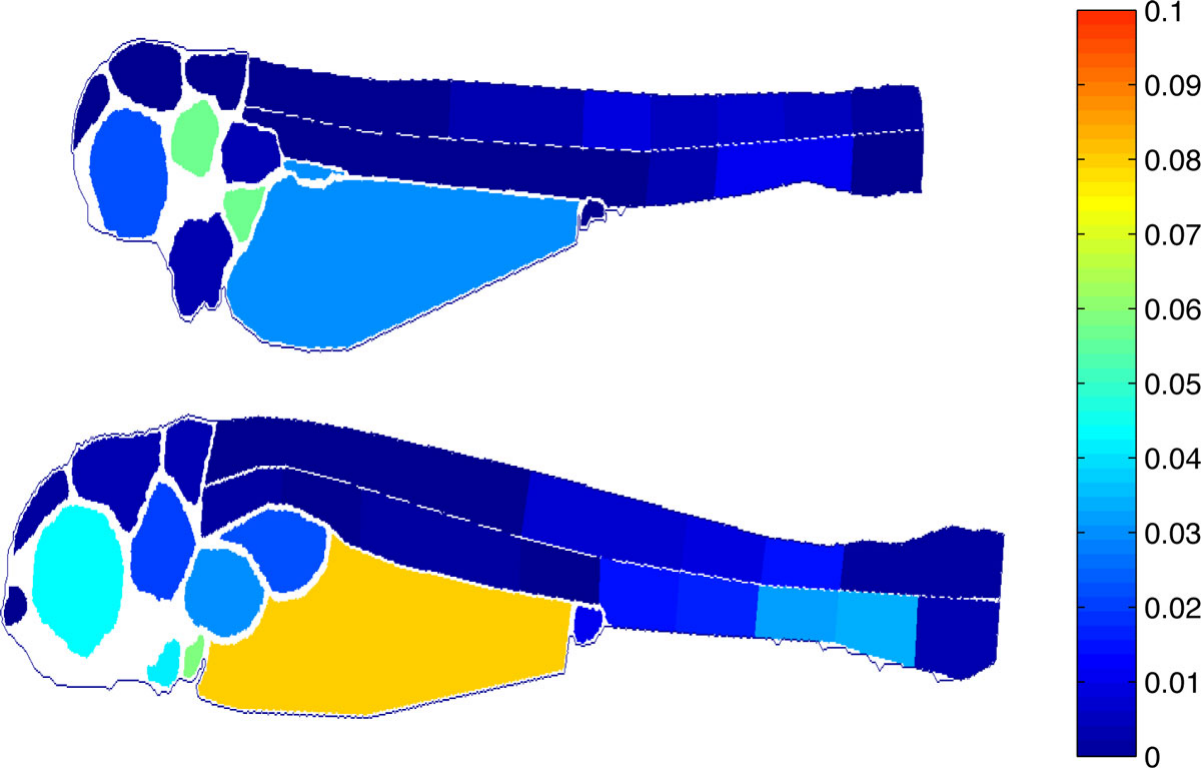
**Area of the cancer spots per organ, normalized by the area of the organ**. Top: Fish at 1 dpi, bottom: Fish at 2 dpi.

Finally, to demonstrate the utility of ZebIAT in extracting biologically relevant information from the images, we show in Figure 7, for the two first days post-injection, the mean fraction of the area of each organ that exhibited cancer spots in the embryos examined. Note that we sub-divided the ventral and dorsal regions into smaller sub-regions. By comparing the top and bottom images, it is possible to see the utility of ZebIAT in studying cancer progression, both in individuals, as well as when averaged over many individuals. For example, from this particular comparison we observe an increase of the areas with cancer cells with time in both the ventral region and yolk. It is also visible a decrease of these cells in, e.g., the otic vesicle, which demonstrates that they migrate throughout the body [1]. Using ZebIAT, not only could one obtain a quantified assessment of this process, but one could also execute a comparative analysis of this process, e.g., for different cancer cells.

## Conclusions

ZebIAT is useful in segmenting and registering embryos of zebrafish at several stages of development, including the internal organs and/or areas of interest selected by a user. ZebIAT performs a temporal, quantitative analysis of the migration of cancer cells in individual fish, and can then produce average results from the analysis of multiple fish. This allows for a comparative analysis between individual fish, or between measurements in different or identical conditions. Its major improvement relative to previous tools, such as [7], is that it allows for an analysis on a per-organ or region basis. This is of significant relevance when using zebrafish as a model of cancer progression as it allows understanding how cancer cells migrate over time.

The automatic landmark extraction was implemented to reduce to a minimum the necessary number of manual landmarks. Since the automatic extraction of landmarks is executed along the contour of each fish, the best accuracy is achieved near the outline of the fish. To register internal organs that may pose particular difficulties, a user interface was created to allow users to add or remove landmarks, following the introduction of the automatically extracted landmarks. Smaller organs are the most prone to errors using automatic landmarks alone, as they may not be fully visible in all frames, which makes it more difficult to find the correspondence between images.

In this study, we did not perform a detailed quantitative analysis of the effects of noise in the land-mark extraction process, since all images had relatively low noise and were obtained following the same methodology. However, we observed that if the fluorescence signal is weak, noise may become problematic. Thus, while the Gaussian filter (in LoG) should suppress the noise to some degree, we recommend that the fluorescence images have sufficient exposure time to reduce noise.

In the future it should be possible to further reduce the need for manual landmarks, particularly, in the organs. We attempted to add automatic landmarks within the fish internal structure, based on feature matching between embryos. However, we failed to find landmarks as robust as those in the outline, due to a higher degree of morphologic variability of the organs between embryos. In any case, provided the identification of more reliable features in the inner organs, this should be feasible. On the other hand, fully automated methods will likely require setting stricter rules on how images should be acquired. In that sense, we believe that ZebIAT has, at the present stage, a good compromise between automated methods and user intervention. Another future improvement that would be of use is to extent the registration methods to 3D-images. The methods used here were selected with this aim in mind. Such extension should help in detecting cancer metastases that extravasate from blood vessels. Moreover, it should prevent some registration errors that may arise from having different angles of rotation along the major axis in each fish.

## List of abbreviations

TPS: thin plate splines, dpf: days post fertilization, DIC: differential interference contrast, GFP: green fluorescent protein, dpi: days post injection, LoG: Laplace of Gaussian

## Competing interests

The authors declare that they have no competing interests.

## Authors' contributions

AR conceived the study. TA and EL designed and implemented the software code, AR, TA and EL wrote the manuscript. IM and DW acquired the data. All authors performed research, and read and approved the final manuscript.

## Declarations

The publication costs for this article were funded by the Finnish Funding Agency for Technology and Innovation (grant no. 40226/12).

This article has been published as part of BMC Bioinformatics Volume 14 Supplement 10, 2013: Selected articles from the 10th International Workshop on Computational Systems Biology (WCSB) 2013: Bioinformatics. The full contents of the supplement are available online at http://www.biomedcentral.com/bmcbioinformatics/supplements/14/S10
